# Scalable synthesis of BiVO_4_ thin films via anodic plating and thermal calcination

**DOI:** 10.1186/s11671-023-03774-z

**Published:** 2023-02-08

**Authors:** Haoyang Jiang, Yongcheng Xiao, Miao Zhong

**Affiliations:** College of Engineering and Applied Sciences, Nanjing University, 163 Xianlin Avenue, Qixia District, Nanjing, 210023 China

**Keywords:** Anodic plating, Thin film, Bismutite, Bismuth vanadate, Solar hydrogen generation

## Abstract

Fabrication of high-quality semiconductor thin films has long been a subject of keen interest in the photocatalytic field. Here, we report a facile, solution-based anodic plating and calcination for large-scale synthesis of BiVO_4_ thin films on indium tin oxide coated glass for use as photoanodes in solar water splitting. Using Na_2_SO_3_ as a sacrificial reagent, continuous solar H_2_ production with 94% Faradaic efficiency was obtained over 6 h of photoelectrochemical water splitting.

**Supplementary Information:**

The online version contains supplementary material available at 10.1186/s11671-023-03774-z.

## Introduction

Solar-driven photoelectrochemical (PEC) water splitting is a promising route for the large-scale production of renewable hydrogen fuel from water [1–5]. In the past decades, much effort has been made to improve the overall energy efficiency of PEC devices [6–9]. In terms of the photocathodes, high photocurrent densities with low overpotentials have been realized using *p*-type solar-cell materials in combination with hydrogen-evolution co-catalysts. However, the improvement in photoanodes remains limited [10].

Among a range of photoanodic materials, BiVO_4_ has attracted research attention because it has a deep valance band position for the oxygen evolution reaction [11]. Also, BiVO_4_ is relatively stable in neutral aqueous environments (pH 7–9) [12, 13]. Over 100-h PEC water oxidation has been reported using crystalline BiVO_4_ photoanodes [14]. One of the remaining challenges for BiVO_4_ is to increase the photocurrent density under photocatalytic conditions without applying any external electrical potential. To this end, Choi et al. reported the synthesis of nanoporous BiVO_4_ photoanodes in a two-step process using BiOI nanoplates as the precursor [15]. Nanostructure certainly improves the charge separation; however, it also presents a difficulty for fabricating a *p*-*n* junction that is able to cover the entire BiVO_4 _to make stand-alone, photocatalytic water-splitting catalysts.

In this work, we report a facile synthesis of BiVO_4_ thin films on transparent, conductive indium tin oxide (ITO) substrates using anodic plating and thermal calcination. A homogeneous mixture of the anodically deposited bismutite hydrate ((BiO)_4_(OH)_2_CO_3_) and vanadium ions (Fig. S1 in the supporting information) allows nucleation of stichometrical BiVO_4_ during calcination. Also, bismutite hydrate decomposes at temperatures > 500 °C and releases CO_2_; the synthesized BiVO_4_ is thus free of contamination. Using Na_2_SO_3_ as a sacrificial reagent, stable photoelectrochemical H_2_ generation was realized over 6 h of water splitting. The present study shows a promising solution-based process for the preparation of BiVO_4_ thin films for use in water-splitting applications.

## Results and discussion

Layer-structured bismutite and its hydrate were first reported in 1943 [16] and systematically studied in 1984 [17]. In mineralogy, bismutite is a well-established solid carbonate in the system Bi_2_O_3_-CO_2_-H_2_O [16] with a natural color of yellow to brown. In the laboratory, the synthesis of bismutite has only been reported using the hydrothermal method and with the products in the form of particles [17, 18]. In the present study, we found that anodic plating can also synthesize amorphous Bi_4_O_4_(OH)_2_CO_3_ thin films on ITO glass via Kolbe electrolysis with the presence of Bi ions, following the Eq. 1:

1 $${\text{CH}}_{3} {\text{COONa}} + {\text{Bi}}({\text{NO}}_{3} )_{3} + {\text{H}}_{2} {\text{O}} \mathop{\longrightarrow}\limits^{{ + 2.3 V_{{{\text{Ag}}/{\text{AgCl}}}} }}({\text{BiO}})_{4} ({\text{OH}})_{2} {\text{CO}}_{3} + {\text{NaNO}}_{3}$$

**Fig. 1:**
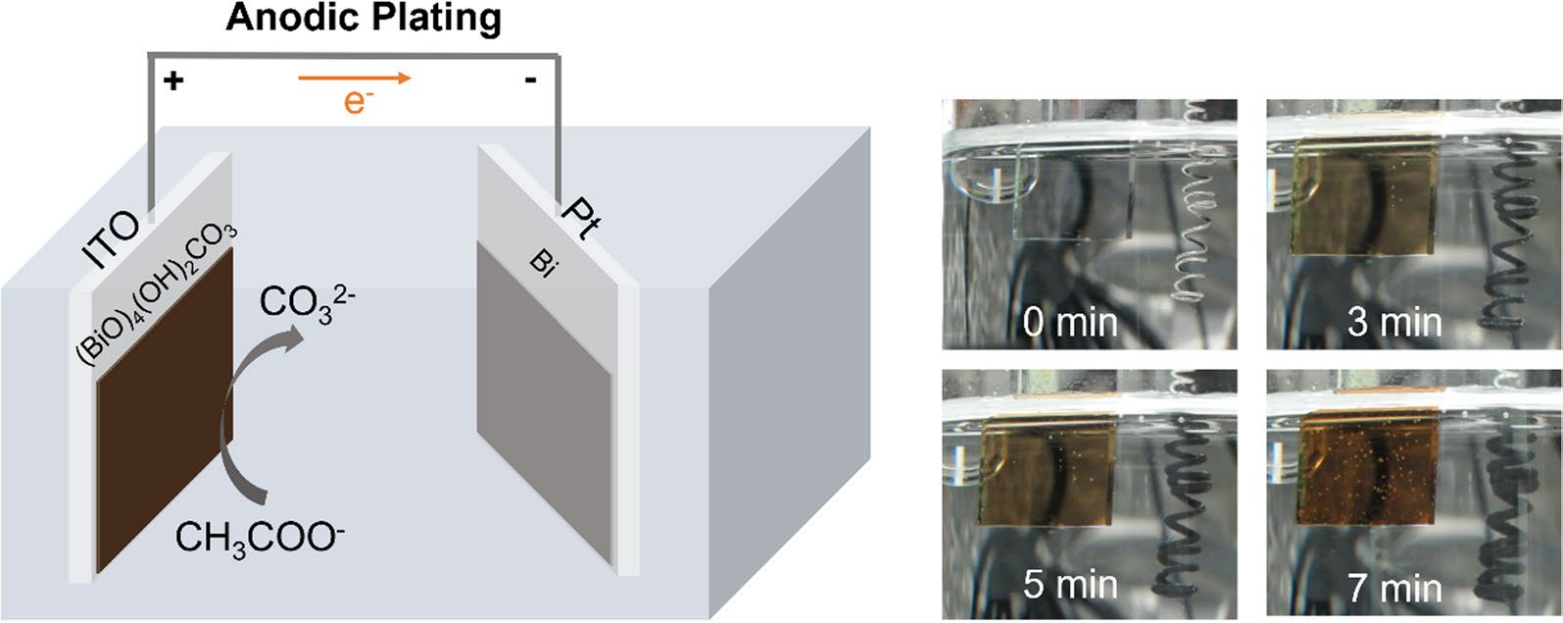
Schematic of the anodic plating for depositing (BiO)_4_(OH)_2_CO_3_ films on ITO glasses

Figure 1 shows the anodic plating of amorphous Bi_4_O_4_(OH)_2_CO_3_ films on ITO substrates using NaCOOH and Bi(NO_3_)_3_ solutions at pH ~ 5. The applied potential is + 2.3 V vs Ag/AgCl (V_Ag/AgCl_). After 7-min anodic plating, ~ 300-nm-thick film was plated. Likely, the NaCOOH was oxidized at anodic potentials (Eq. 1) to form CO_2_ on the electrode surfaces. With the presence of Bi^3+^ in the solution, Bi_4_O_4_(OH)_2_CO_3_ precipitated on the electrodes. We also found that Bi metals precipitated on the cathode. This is because the reduction potential of Bi^3+^ to Bi was + 0.2 V_RHE_, which is more positive than 0 V_RHE_ of the hydrogen evolution reaction. To suppress the Bi precipitation, *p*-benzoquinone can be added to the electrolyte. The cathodic reaction then mainly shifts to the reduction of *p*-benzoquinone to 1,4-hydroquinone with a reduction potential of ~  + 0.6 V_RHE_ (Fig. S2). The optical images of the anodically plated amorphous Bi_4_O_4_(OH)_2_CO_3_ films are provided on the right panel in Fig. 1, which shows the change of color at the different time of the plating.

**Fig. 2:**
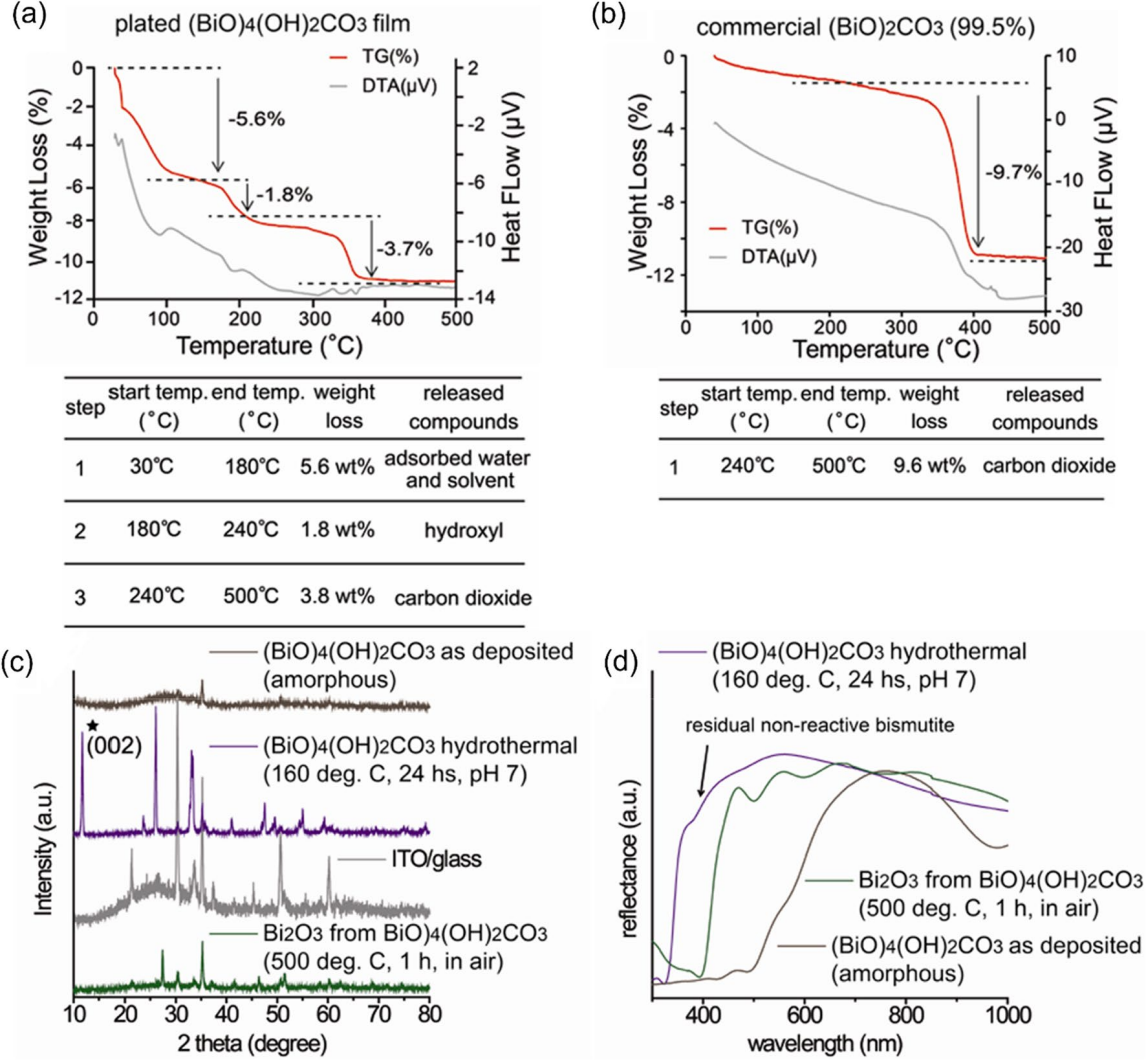
**a** TG and DTA of a plated (BiO)_4_(OH)_2_CO_3_ film. **b** TG and DTA of the commercial (BiO)_2_CO_3_ (99.5%). **c** XRD pattern of the as-plated (BiO)_4_(OH)_2_CO_3_, hydrothermal-treated (BiO)_4_(OH)_2_CO_3_, ITO and Bi_2_O_3_ calcinated from (BiO)_4_(OH)_2_CO_3_. **d** UV–vis diffuse reflectance spectra of the as-plated (BiO)_4_(OH)_2_CO_3_, hydrothermal-treated (BiO)_4_(OH)_2_CO_3_, ITO and Bi_2_O_3_ calcinated from (BiO)_4_(OH)_2_CO_3_

**Fig. 3:**
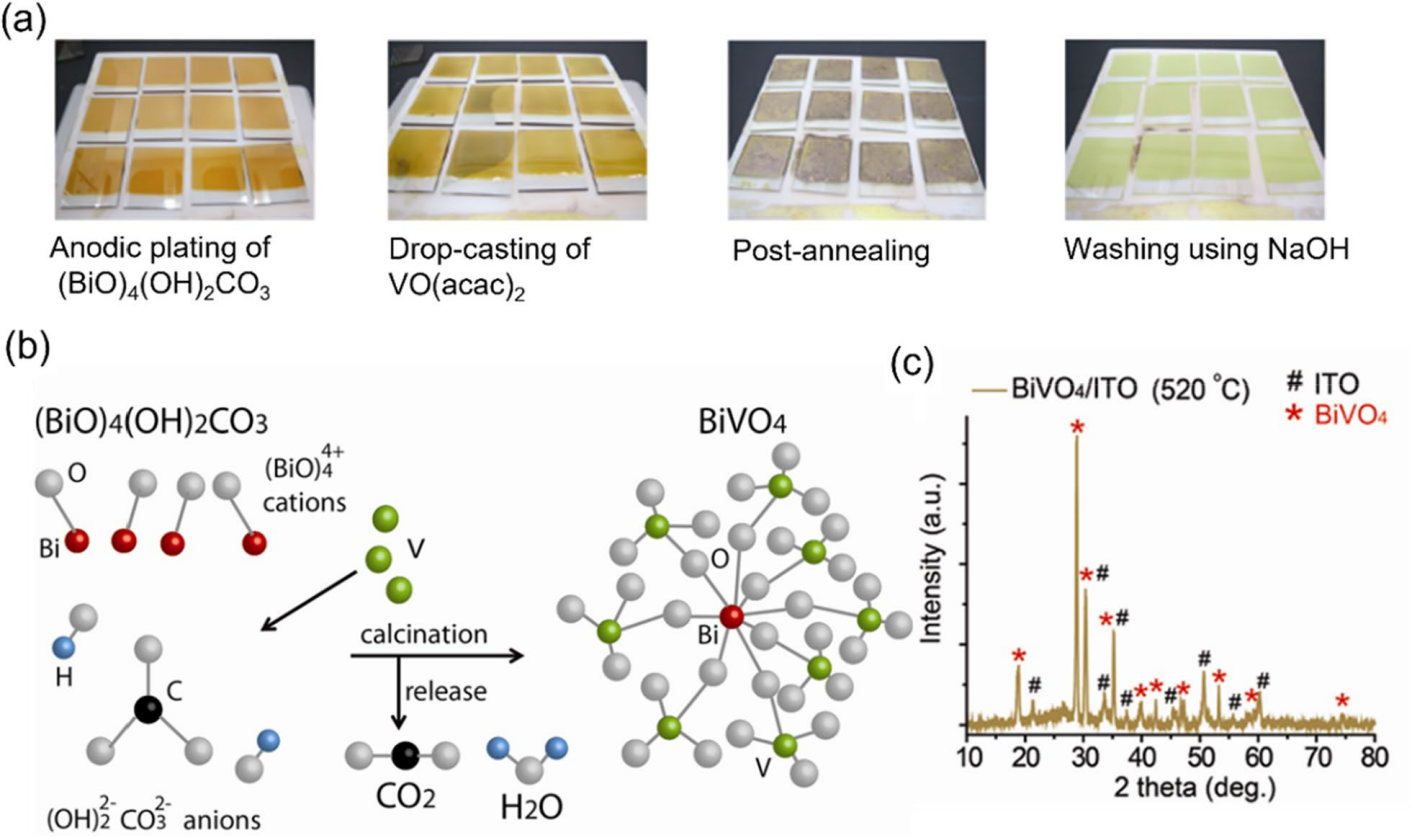
**a** Optical images of the solution-based process for the synthesis of BiVO_4_ films on ITO glass (12 pieces of the 2 cm × 2 cm samples). **b** Schematic diagram of  a proposed reaction mechanism. **c** XRD patterns of the BiVO_4_/ITO synthesized at 520 °C

To understand anodic plating details, Bi_4_O_4_(OH)_2_CO_3_ films were characterized by scanning electron microscopy (SEM), X-ray photoelectron spectroscopy (XPS), X-ray diffraction (XRD), UV–Vis diffuse-reflectance analyses and thermogravimetric analysis (TGA) (Figs. 2, 3 and Figs. S3, S4 in the supporting information). To evaluate constituent compositions, the plated Bi_4_O_4_(OH)_2_CO_3_ films were scratched from ITO/glasses for TGA with a temperature rise from 30 to 500 °C in an N_2_ atmosphere (N_2_ was used to avoid adsorption of CO_2_ from the air). As shown in Fig. 2a, three steps of endothermic decomposition were obtained in a TGA run for Bi_4_O_4_(OH)_2_CO_3_. A continuous weight loss of 5.6% below 180 °C was observed, likely attributed to absorbed solvent and water [17]. Bi_4_O_4_(OH)_2_CO_3_ decomposition often occurs in two stages [17]: major loss of hydrate with a small loss of carbon dioxide at 180–240 °C and major loss of carbon dioxide at 240–500 °C. The calculation of weight losses in each step yielded an empirical formulation of (BiO)_4_(OH)_1.01_(CO_3_)_0.94_ of the anodically plated films, which agreed with the predicted products of Bi_4_O_4_(OH)_2_CO_3_. As a reference, we also analyzed Bi_2_O_2_CO_3_ powder (Wako, 99.5%) by TGA under the same conditions. Decomposition to release carbon dioxide was observed at 240–500 °C with a weight loss of 9.6 wt%, close to the theoretic value of 8.6 wt%. A slight shift of decomposition onset temperature of Bi_2_O_2_CO_3_ compared to that of plated Bi_4_O_4_(OH)_2_CO_3_ films was likely due to the crystalline and amorphous nature of the two materials. This result suggested that amorphous Bi_4_O_4_(OH)_2_CO_3_ films were anodically plated on ITO.

Figure 3a shows the optical images of the synthesis process of BiVO_4_ thin films on ITO substrates. In brief, we deposited amorphous Bi precursors on ITO substrates and calcinated them with the vanadium source at 520 °C. After the reaction, we washed the surface residual vanadium chemicals using 1 M NaOH solution. This process was similar to our previous report, in which the BiVO_4_ was fabricated via three steps: precursor deposition, pre-calcination of the deposited films in the air at 200 °C, and calcination with a vanadium source at 490–530 °C. We used two-step fabrication, which was able to fabricate BiVO_4_ thin films with similar morphology. The Bi precursor materials were calcined in the air at 520 °C (Fig. 3c) for 2 h. The fabricated BiVO_4_ films were in a monoclinic structure, which agreed with the previous report [8].

**Fig. 4:**
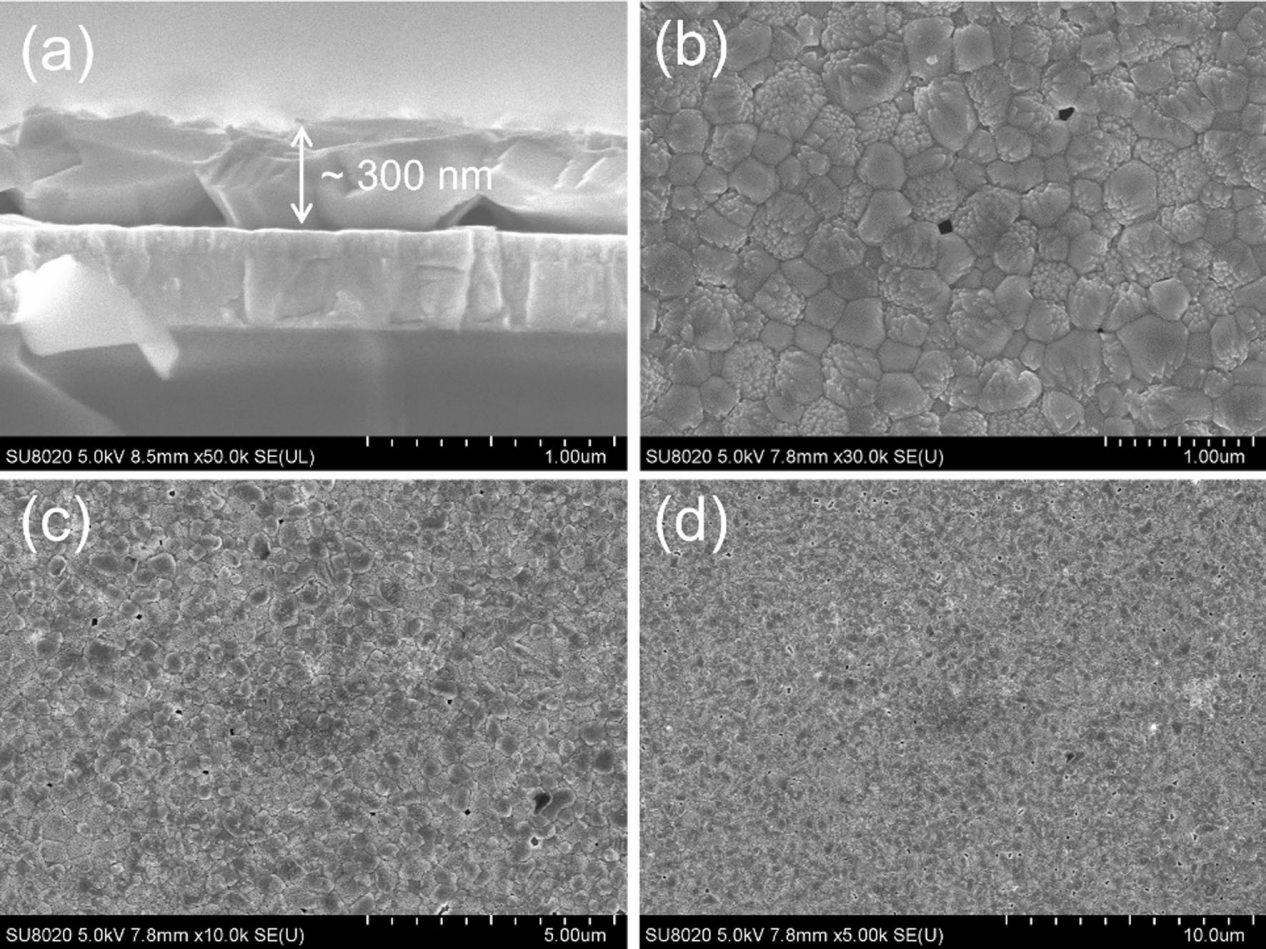
SEM images of BiVO_4_. **a** The cross-sectional view of BiVO_4_. **b**–**d** The top view of BiVO_4_

The scanning electron microscopy (SEM) images of the plated Bi_4_O_4_(OH)_2_CO_3_ film are present in Fig. S3 in the supporting information. The 7-min anodically plated amorphous Bi_4_O_4_(OH)_2_CO_3_ film was ~ 300 nm thick on the ITO substrate (Fig. S3). Bi was detected on the surface and in the bulk of the film (Fig. S4). As shown in cross-sectional SEM images in Fig. 4, the BiVO_4_ film was made of large BiVO_4_ particles with an in-plane diameter of ~ 500–1000 nm and a thickness of ~ 300 nm. Such large BiVO_4_ crystalline likely decreased the number of boundaries between particles. Therefore, improved photoelectrochemical performance was realized.

**Fig. 5:**
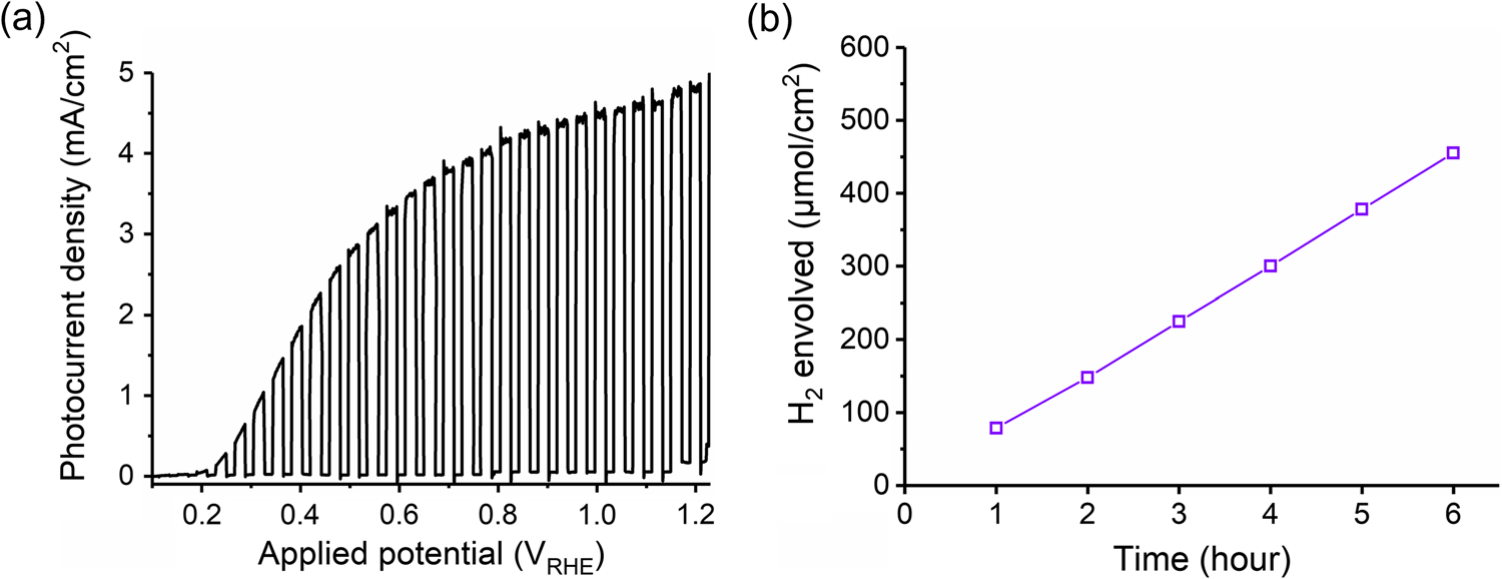
Photoelectrochemical performance of BiVO_4_ film in a 0.1 M potassium phosphate (KPi) and 0.1 M Na_2_SO_3_ solution. **a** Photocurrent densities at 0–1.2 V_RHE_; **b** evolved H_2_ in 6 h

Finally, we tested the photoelectrochemical performance of the fabricated BiVO_4_ films in 0.1 M Na_2_SO_3_ solution. As shown in Fig. 5a, a quick raise of the photocurrent density was observed with BiVO_4_ films with the increase of the positive potential. At 0.9–1.2 V_RHE_, the photocurrent density reached a plateau of ~ 5 mA/cm^2^ under simulated solar light irradiation. We used a micro gas chromatography (micro-GC) to analyze the hydrogen evolution, which was stable over 6-h photoelectrochemical water splitting at 0.9 V_RHE_ (Fig. 5b).

## Conclusions

In this work, anodic plating was reported for the fabrication of Bi precursors on the indium tin oxide (ITO) substrates. Following high-temperature calcination with vanadium sources, crystalline BiVO_4_ was fabricated on ITO substrates. The photocurrent density reached ~ 4–5 mA/cm^2^ at 0.9–1.2 V_RHE_ in Na_2_SO_3_-containing electrolytes under simulated solar illumination. The developed electrochemical deposition and thermal calcination may offer a new pathway for the synthesis of photocatalytic materials.

## Experimental

### Anodic-plating of Bi_4_O_4_(OH)_2_CO_3_ film on ITO glass

A 0.1 M Bi(NO_3_)_3_ solution was prepared by dissolving Bi(NO_3_)_3_·5H_2_O in 25 ml acetic acid solution (99.7% mass ratio, Wako). The prepared solution was mixed with a 10 mL pH 5 sodium acetate aqueous solution. 5 M NaOH solution was used to adjust the pH of the mixed solution to 4.8. Anodic plating was performed at 2.3 V_Ag/AgCl_ at room temperature using a three-electrode cell with ITO working electrodes, a platinum counter electrode and an Ag/AgCl reference electrode. A potentiostat (CHI630e) was used for anodic plating and subsequent electrochemical measurements. The optimum plating time was 7 min.

### Fabrication of BiVO_4_ thin film on ITO glass

First, ~ 300 nm amorphous Bi_4_O_4_(OH)_2_CO_3_ film was anodically plated on an ITO glass in a bismuth nitrite and sodium acetate aqueous solution. Drop-casting vanadyl acetylacetonate (VO(acac)_2_) organic solutions onto the plated Bi_4_O_4_(OH)_2_CO_3_ films allowed homogeneous mix of the V and Bi species. The mixed samples were annealed at different temperatures. Finally, the obtained film samples were washed with NaOH to remove impurities. In detail, 0.075 mL 0.2 M VO(acac)_2_ dimethyl sulfoxide solution was dropped on the Bi_4_O_4_(OH)_2_CO_3_ films (2 cm × 2 cm) and then calcined in a furnace at 520 °C for 2 h in air. Remained VO_*x*_ on top of the BiVO_4_ films was washed in 1 M NaOH solution for 10 min with gentle stirring.

### Measurements

The scanning electron microscopic (SEM) images were obtained by a Hitachi SU8020. The XRD diffraction spectra were performed using the smart lab XRD of Rigaku, Japan. The XPS analyses were performed using Mg Kα (1253.6 eV) photon energy. During XPS depth profile studies, Ar bombardment with an etching speed of several tens nm/time was used. Binding energy peak shifts due to any charging were normalized with C 1s peak set to 284.8 eV and Fermi energy position. The TGA analyses were conducted with a differential thermogravimetric analyzer (Rigaku, Japan). The PEC performances were measured by a three-electrode electrochemical configuration with a 0.1 M Na_2_SO_3_ solution under simulated sunlight illumination (SAN-EI electronic, XES40S1, AM 1.5G, 100 mW cm^−2^). An Ag/AgCl electrode was used as a reference electrode, and a Pt coil was used as a counter electrode. The measured potentials were all converted to the reversible hydrogen electrode according to the Nernst equation:

2 $$V_{{{\text{RHE}}}} = V_{{{\text{Ag}}/{\text{AgCl}}}} + 0.059\,{\text{pH}} + V_{{{\text{Ag}}/{\text{AgCl}}}}^{0}$$

3 $$V_{{{\text{Ag}}/{\text{AgCl}}}}^{0} = 0.199\,{\text{V}}\,{\text{at}}\,25\,^{ \circ } {\text{C}}$$

The PEC cell was connected to a vacuum pump and a micro-GC (Agilent 990 micro). Before measurement, the PEC cell was pumped to a low vacuum, and Ar gas was used to purge out the N_2_ and O_2_ gases in the cell. The H_2_ evolution was measured in under simulated sunlight illumination for 6 h at 0.9 V_RHE_. The theoretical amounts of evolved H_2_ were estimated from the passed charges on the assumption that faradaic efficiency was unity.

## Supplementary Information

Supplementary file1 (DOCX 3034 KB)

## Data Availability

The datasets used and/or analyzed during the current study are available from the corresponding author on reasonable request.

## Abbreviations

ITO: Indium tin oxide
PEC: Solar-driven photoelectrochemical
SEM: Scanning electron microscopy
XPS: X-ray photoelectron spectroscopy
XRD: X-ray diffraction
TGA: Thermogravimetric analysis
