# Supplementary material for: Scalable synthesis of BiVO4 thin films via anodic plating and thermal calcination
Source: Discov Nano. 2023 Feb 8;18(1):6. doi: 10.1186/s11671-023-03774-z (PMC9908786; doi:10.1186/s11671-023-03774-z)
Supplement: Supplementary file 1 — Supplementary file1 (DOCX 3034 KB) [file 11671_2023_3774_MOESM1_ESM.docx]

Supporting Information

Scalable synthesis of BiVO_4_ thin films via anodic plating and thermal calcination

Haoyang Jiang, Yongcheng Xiao, Miao Zhong*

* Corresponding author: miaozhong@nju.edu.cn

College of Engineering and Applied Sciences, Nanjing University, 163 Xianlin Avenue, Qixia District, Nanjing 210023, China


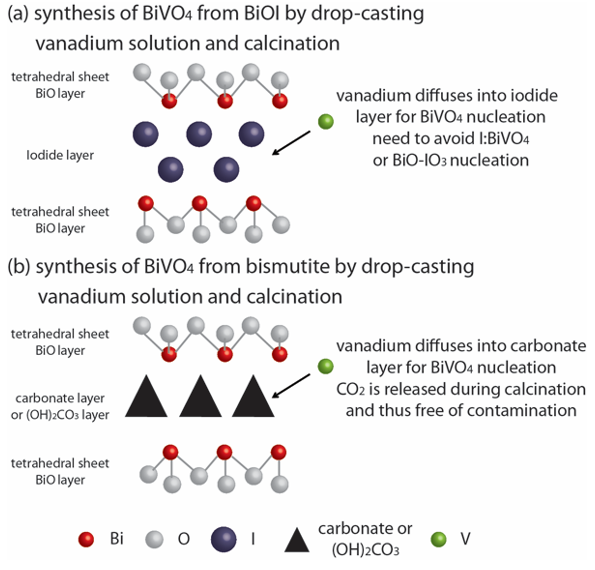


**Fig. S1.** Schematic of BiOI (a) and bismutite hydrate (b) layered structures for uniform mixing of vanadium and BiO by vanadium diffusion through iodide and carbonate layers.


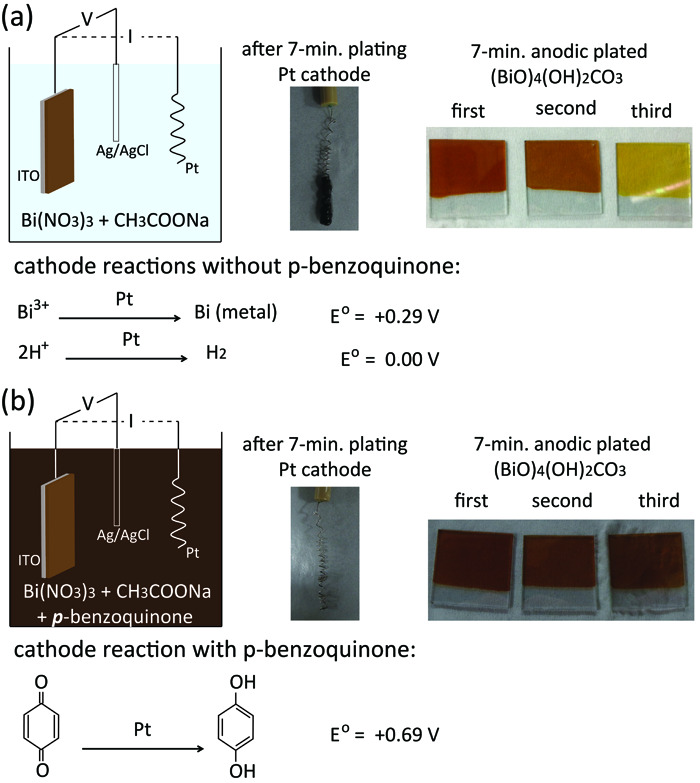


**Fig. S2.** Plating of Bi precursor films with and without adding sacrificial reagent.

To avoid Bi precipitation on the cathode side during wet-plating Bi_4_O_4_(OH)_2_CO_3_ films, 20 mL 0.3 M *p*-benzoquinone in absolute ethanol (99.95%) solution was added to the plating solution. The pH was raised to 5.3 after adding *p*-benzoquinone and the obtained solution was gently stirred for one hour until its colour was transparent black.


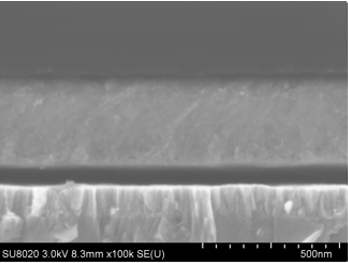


**Fig. S3.** SEM of Bi_4_O_4_(OH)_2_CO_3_ films.


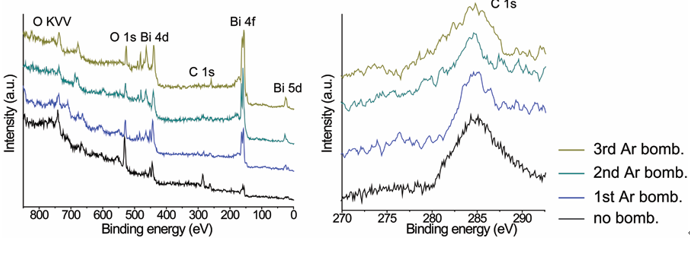


**Fig. S4.** XPS depth profile analyses of the plated (BiO)_4_(OH)_2_CO_3_ film with soft Ar bombardment.
